# Evolution of beverage portion sizes consumed in Brazil between 2008 and 2018

**DOI:** 10.3389/fpubh.2022.969045

**Published:** 2023-01-11

**Authors:** Jessica Brito Cavalcante, Thais Meirelles de Vasconcelos, Rosely Sichieri, Ilana Nogueira Bezerra

**Affiliations:** ^1^Programa de Pós-Graduação em Nutrição e Saúde, Centro de Ciências da Saúde, Universidade Estadual do Ceará, Fortaleza, CE, Brazil; ^2^Programa de Pós-Graduação em Saúde Coletiva, Centro de Ciências da Saúde, Universidade Estadual do Ceará, Fortaleza, CE, Brazil; ^3^Departamento de Epidemiologia, Instituto de Medicina Social, Universidade Estadual do Rio de Janeiro, Rio de Janeiro, RJ, Brazil

**Keywords:** food consumption, diet records, beverages, portion sizes, data collection

## Abstract

**Objective:**

To describe the evolution of beverage portion sizes consumed in Brazil between 2008 and 2018.

**Methods:**

Data from the dietary surveys of 2008–2009 and 2017–2018 Brazilian Household Budget Surveys, conducted with 34,003 and 46,164 individuals, respectively, were used to analyze the portion size of beverages. Food consumption data were used to group beverages according to nutritional characteristics, type and size of portions into eight groups: high-calorie soft drinks, fruit refreshments, alcoholic beverages, coffee/tea, fruit juices, milk and milk substitutes and ultra-processed milk-based beverages. The two-day food record and recall were considered to analyze the consumed portions. Comparisons between the surveys were done using Chi-Square tests and linear regression models.

**Results:**

Between 2008 and 2018, the average portion consumed showed a significant increase for the group of alcoholic beverages (+29%), flavored juices (+11%), caloric soft drinks (+8%), milk and milk substitutes (+6%) and fruit juices (+5%); and reduction for the coffee/tea group (−11%). Analyzes by age group showed that among individuals between 20 and 40 years of age, the soft drinks and alcoholic beverage groups showed the greatest increase in portion size, +12 and +44%, respectively.

**Conclusion:**

The beverage portion sizes consumed in Brazil between 2008 and 2018 increased for the group of alcoholic beverages, flavored juices, caloric soft drinks, milk and milk substitutes, and fruit juices.

## 1. Introduction

The consumed amount of certain foods has been increasing in recent years alongside obesity rates. There is evidence that exposure to large portions of high-calorie foods leads to increased energy intake, agreeing with the phenomenon of the portion size effect (1). There is evidence that when the portion of food is doubled, there is a 35% increase in the total energy intake, which may favor excessive weight gain in both adults and children (2). These findings are even more important when it comes to consumption of beverages rich in free sugars, such as soft drinks and artificial fruit juices. The reduced satiety provided by these beverages seems to induce excessive energy intake and promote overweight, obesity, diabetes and cardiovascular disorders (1).

A trend toward an increase in the size of the beverage portions has been observed in several countries (2–5). There is evidence that individuals drink significantly more when using a larger-sized cup (18 vs. 12 oz.) (6). In the United States, the average serving size of sugar-sweetened beverages more than tripled in volume between the 1950s and 2010 (192 vs. 591 ml, respectively) (7).

Although there is evidence that the increase in portion sizes is one of the factors stimulating the growing increase in overweight and obesity, as well as related diseases, there are no studies for the Brazilian population that demonstrate changes in food portion sizes over the years. The first national dietary survey in the country was carried out in 2008–2009 and the second one in 2017–2018, and this is the first time that Brazil has data on individual food consumption in a representative sample of its population (8, 9).

Understanding the factors that induce individuals to consume more than usual and how they react to these stimuli may be the key to reducing the prevalence of obesity, as well as producing data to support public policies to fight obesity (4). The objective of this study was to describe the evolution of beverage portions consumed in Brazil between 2008 and 2018, using data from national dietary surveys.

## 2. Materials and methods

### 2.1. Study design and sample

The study used data of the Brazilian National Survey conducted along with the Brazilian Household Budget Surveys (HBS) from 2008–2009 and 2017–2018. Both editions have sampling plans by conglomerate in two stages, with the selection of the population from IBGE (Brazilian Institute of Geography and Statistics) Integrated System of Household Surveys, which has a broad common sample representative of the Brazilian population.

This common sample follows a complex two-stage sampling scheme, where the primary sampling units were the census sectors selected by probability proportional to the number of households in the strata. The census sectors were previously obtained by geographic and income stratification based on the Demographic Census of the year 2000 for the HBS 2008–2009 and of the year 2010 for the HBS 2017–2018. In the second stage, the sampling units were the households selected by simple random sampling. Then, for the assessment of food consumption, a subsample of households was selected from the main sample by simple random sampling.

In total, for the HBS 2008–2009, 4,696 sectors were selected and 55,970 households were interviewed, with the participation of 34,003 individuals in the food consumption module. The HBS 2017–2018 consisted of 5,504 elected sectors and 57,920 investigated households, with the participation of 46,164 individuals in the food consumption module. For this article, only individuals who answered the 2 days of food consumption were included, comprising 32,900 individuals in 2008–2009 and 38,854 individuals in 2017–2018.

Data collection was carried out by trained IBGE agents through interviews with residents over 10 years of age in selected households. In all, it took 9 days to complete seven questionnaires. The collected information was stored in a data inclusion program developed by IBGE. For this research, information from the modules that investigated demographic data (age and sex), as well as from the food consumption module were used.

### 2.2. Food consumption data

In the HBS 2008–2009, food consumption data was collected through food records. The interviewees received advice on how they should fill out the individual food consumption data. The recording took place on two non-consecutive days and interviewers recorded in the notebook all the foods consumed, including sweeteners and/or sugar.

For the 2017–2018 edition of the HBS, food consumption data was collected through 24-h recall, also used on two non-consecutive days, with the instrument being filled out by a trained agent who personally interviewed the individual. Food and beverages, including water, consumed the day before the visit, were investigated and recorded following the script structured in sequential stages based on the Multiple-Passage Method (10). The list was first written on paper to capture the food items and then transferred to the tablet containing a program developed especially for this evaluation. The software, in addition to contemplating the items investigated in the previous edition, detailed the “addition items,” which are usually omitted. Addition items and water consumption were not included in the analyses as they were not collected in the 2008–2009 survey.

The changes in data collection between the surveys were detailed and explained in a previous publication. The major reasons for the changes were due to the lower probability of systematic error of 24 h-recalls in population surveys and validation study showed better performance of the 24 h-R compared to the food record (REF).

In both editions, information was included about the type of preparation for specific foods, such as beef, chicken, fish, egg, and some vegetables, the time and place of consumption, and the final amount consumed. For all consumed foods, individuals reported the amount consumed using home measures (cups, glasses, and household tableware) to facilitate the correct filling of the amount of food consumed. They also received a booklet with photos of kitchen utensils and containers most used to serve food, in order to help estimate intake quantities. The consumption of sugar and/or sweetener was questioned in both surveys, asking the individuals what they used most often to sweeten their beverages. At the end of the food record or after the application of the recall, the trained agent verified the omission of frequently underreported items and solved doubts about any unusual information.

To estimate the consumed amount, a table of measures related to food consumed in Brazil from the HBS 2017–2018, with their respective amounts in grams or milliliters, was prepared based on an extensive review and update of the table of measures related to the foods consumed in Brazil from the HBS 2008–2009. The previous methodology was maintained, with the compilation of Brazilian household measurements and other sources of information, such as: publications containing information on the weight of household measurements; food labels; scientific articles containing the unit weight of some national fruits; and direct weighing of some foods and preparations carried out in research centers at Brazilian universities.

### 2.3. Statistical analysis

For the analysis of the consumed portions, the 2 days of recording and food recall were considered. First, all beverages mentioned in the survey were grouped according to their nutritional characteristics and consumption modes into eight groups: high-calorie soft drinks, fruit refreshments, alcoholic beverages, coffee/tea, fruit juices, milk and milk substitutes, and ultra-processed milk-based beverages. The description of each group is presented in Table 1.

**Table 1:** Frequency (%) and 95% confident interval (95% CI) of consumers of beverages groups in Brazil in 2008–2009 and 2017–2018.

**Beverage groups**	**Group description**	**2008–2009**	**2017–2018**
High-calorie soft drinks	Traditional carbonated, sports and energy drinks with added sugar	35.2 (33.8–36.5)	25.2^*^ (24.1–26.2)
Fruit refreshments	Industrialized beverages, with low fruit juice content, which can be the traditionally sweetened powder (sugar) or not (diet/light)	12.1 (11.2–13.0)	7.9^*^ (7.3–8.6)
Alcoholic beverages	Distilled and fermented beverages with an alcohol content, which may or may not be added to fruits and/or other ingredients	6.6 (6.1–7.2)	6.3 (5.8–6.8)
Coffees/teas	Drinks with caffeine or made of the infusion of leaves, flowers, plant roots, traditionally sweetened (sugar) or not (diet/light)	86.4 (85.5–87.3)	87.8^*^ (87.1–88.6)
Fruit juices	Beverages extracted from the fruit, which may or may not be pure/diluted, sweetened or organic	46.1 (44.7–47.4)	46.6 (45.4–47.8)
Milk and milk substitutes	Skimmed, whole or semi-skimmed cow's milk	25.8 (24.7–26.8)	14.9^*^ (14.1–15.6)
Ultra-processed milk-based beverages	Milk-based beverages that are generally ready for consumption, with added chocolate or food supplement or fermented	15.2 (14.2–16.1)	10.6^*^ (9.9–11.3)

Household Budget Survey 2008–2009 and 2017–2018.

^*^p-value < 0.05 for χ^2^ Test.

Aiming at standardizing the analysis of liquid beverages, when the individual did not know the dilution used to prepare the beverage, we used the usual concentrations indicated by the manufacturer and the net volume for household measurements related to the powder was estimated (0.2% of reports in both surveys). The powdered drinks, before preparation, were: brewer's yeast, powdered barley, powdered cocoa, chocolate-flavored powder, diet shake, whole and skimmed milk powder, powdered soy milk.

The portion size was estimated among the individuals who consumed at least one item from the beverage group in one of the two food record days, being defined as the total consumed amount of all items from the beverage group (in mm) divided by the number of occasions when those items were consumed. This methodology was proposed by McConahy et al. (11) and Huang et al. (12) and used in a previous Brazilian study (13) that investigated only the 2008–2009 period. The energy density of each group was calculated by dividing the energy content (in kcal) by the weight of beverages (in ml) consumed. I'm estimate the energy content, the Brazilian Food Composition Table (Brazilian Food Composition Table-TBCA version 7.0) was used (9). The TBCA is available at http://www.tbca.net.br/ 12 and it is adequate to the national survey context (14).

The percentage of individuals who consumed each beverage group and the median of the consumed portion of each group were estimated. The average portion size of the consumed beverage groups was estimated among individuals who recorded the consumption of at least one item from the group in the two food record days. Estimates were generated according to age group (10–14 years old, 15–19 years old, 20–39 years old, 40–59 years old, and 60 or more years old) and sex.

All these estimates were calculated for the two surveys. Differences in the frequency of consumers of beverage groups between the surveys were tested through Chi-Square tests (χ^2^). Linear regression models were performed to evaluate differences in the average consumption and energy density of each beverage group, considering the year of the survey as the independent variable. We also observed differences between the 95% confidence intervals. All statistical analyses were performed using SAS software, version 9.1.3, considering sample weights and the effect of the study design of each survey.

This research was carried out based on secondary data, and it was not possible to identify the participants. The data are public and available on the IBGE website, and approval by the Ethics Committee was not required.

## 3. Results

The 10-year trend was a decrease in the percentage of people who mentioned the consumption of beverages in each group, with the exception of alcoholic beverages, coffee/tea and fruit juices, which maintained the same frequency of consumers between the surveys. The three most consumed beverage groups in both editions were: coffee/teas, fruit juices, and high-calorie soft drinks (Table 1).

The consumed portion size in the beverage groups increased for most groups, with the largest increases observed for alcoholic beverages (+29%), fruit refreshments (+11%), high-calorie soft drinks (+8%), milk and milk substitutes (+6%) and fruit juices (+5%). The coffee/tea group was the only one that showed a reduction at the national level (−11%) and in both genders, with a predominant decrease in the female sex (−13%; Table 2).

**Table 2:** Average consumption (in mm) and 95% confident interval (95% CI) of beverages groups by sex, in 2008–2009 and 2017–2018.

**Beverage groups**	**Brazil**		**Men**		**Woman**	
	**2008–2009**	**2017–2018**	**2008–2009**	**2017–2018**	**2008–2009**	**2017–2018**
High-calorie soft drinks	311.0 (304.7–317.2)	335.8^*^ (327.2–344.3)	336.5 (327.4–345.6)	367.5^*^ (353.7–381.3)	285.0 (279.2–290.8)	301.8^*^ (294.4–309.2)
Fruit refreshments	260.5 (253.6–267.3)	288.1^*^ (276.9–299.3)	277.3 (266.7–287.9)	314.4^*^ (295.4–333.4)	245.3 (238.9–251.6)	263.4^*^ (252.4–274.5)
Alcoholic beverages	713.9 (663.9–764.0)	917.6^*^ (847.2–988.0)	781.4 (719.6–843.2)	1009.9^*^ (923.1–1096.6)	533.2 (467.5–599.0)	739.8^*^ (655.5–824.2)
Coffees/teas	178.9 (175.5–182.4)	159.5 (155.5–163.5)	184.2 (180.0–188.4)	168.2^*^ (162.9–173.4)	174.2 (170.8–177.7)	151.8^*^ (147.9–155.6)
Fruit juices	266.7 (263.5–269.9)	280.1^*^ (276.1–284.1)	282.0 (277.4–286.6)	300.1^*^ (294.2–306.0)	253.4 (250.0–256.8)	263.1^*^ (259.4–266.9)
Milk and milk substitutes	236.6 (233.1–240.0)	251.2^*^ (245.6–256.7)	248.8 (243.3–254.3)	265.9^*^ (259.2–272.5)	226.5 (223.0–230.1)	239.7^*^ (232.7–246.7)
Ultra-processed milk-based beverages	211.2 (205.5–216.8)	219.9^*^ (214.8–225.0)	217.7 (209.6–225.7)	226.5 (218.9–234.0)	205.6 (199.1–212.0)	214.0 (208.3–219.7)

Household Budget Survey 2008–2009 and 2017–2018.

^*^p-value < 0.05 for linear regression models, comparing differences between the surveys.

Among the groups of beverages that showed an increase, it is important to highlight the group of alcoholic beverages, which demonstrated a substantial increase in the average portion size both in Brazil (+29%) and in the male sex (+29%), with this percentage being even higher among women (+39%; Table 2).

When dividing by age group, the reduction in the portion size of the coffee/tea group permeated all ages, with a predominant decrease (−14%) in individuals between 15 and 19 years old. The group of high-calorie soft drinks was the second with the highest increase in the portion size between the age groups, with an increase (+12%) in those aged 20–39 years. This same age group also showed a significant increase (+44%) in the consumption of alcoholic beverages in relation to the increase in those individuals aged between 60 or more years old (+35%), in addition to leading the increase (+9%) in the group of milk and milk substitutes. Individuals aged between 40 and 60 years showed a greater increase in portion size for the groups of fruit juices (9%) and fruit refreshments (17%; Table 3).

**Table 3:** Average consumption (in mm) and 95% confident interval (95% CI) of beverages groups by age, in 2008–2009 and 2017–2018.

**Beverage group**	**10–14 years**		**15–19 years**		**20–39 years**	
	**2008–2009**	**2017–2018**	**2008–2009**	**2017–2018**	**2008–2009**	**2017–2018**
High-calorie soft drinks	314.8 (294.2–335.4)	311.0 (297.2–324.7)	324.6 (311.7–337.4)	358.4^*^ (341.7–375.1)	320.1 (311.0–329.1)	358.1^*^ (341.9–374.3)
Fruit refreshments	249.6 (238.7–260.5)	252.8 (239.6–265.9)	277.9 (261.9–293.8)	295.3 (279.6–310.9)	270.5 (260.3–280.8)	298.4^*^ (280.4–316.4)
Alcoholic beverages	375.8 (262.2–489.4)	497.1 (344.8–649.3)	849.5 (658.6–1040.4)	1134.9 (795.4–1474.4)	764.7 (690.6–838.8)	1100.8^*^ (974.4–1227.9)
Coffees/teas	183.3 (177.9–188.7)	159.2^*^ (146.0–172.4)	189.7 (183.4–195.9)	162.2^*^ (153.8–170.7)	178.5 (173.8–183.2)	159.8^*^ (154.0–165.6)
Fruit juices	268.1 (259.1–277.0)	260.3 (254.0–266.7)	277.8 (267.6–287.9)	281.2 (273.4–289.1)	272.4 (267.6–277.2)	290.1^*^ (285.0–295.3)
Milk and milk substitutes	238.7 (231.9–245.5)	245.3 (235.5–255.1)	238.3 (229.9–246.7)	255.7^*^ (244.0–267.4)	241.6 (236.3–246.8)	263.2^*^ (249.0–277.5)
Ultra-processed milk-based beverages	211.8 (204.4–219.3)	225.0^*^ (217.7–232.3)	215.6 (204.3–226.9)	224.0 (212.6–235.3)	215.1 (206.4–223.7)	221.1 (212.9–229.2)
**Beverage group**	**40–59 years**		≥**60 years**	
	**2008–2009**	**2017–2018**	**2008–2009**	**2017–2018**
High-calorie soft drinks	299.3 (289.8–308.7)	318.6^*^ (309.3–327.9)	262.0 (248.8–275.3)	294.2^*^ (282.3–306.2)
Fruit refreshments	253.3 (243.6–263.0)	295.7^*^ (267.3–324.1)	231.6 (220.9–242.3)	265.2^*^ (245.4–285.0)
Alcoholic beverages	711.7 (632.3–791.1)	879.6^*^ (791.1–968.2)	436.3 (364.5–508.1)	587.2^*^ (510.6–663.8)
Coffees/teas	178.1 (172.8–183.4)	159.1^*^ (153.8–164.4)	172.4 (166.2–178.6)	158.6^*^ (152.8–164.4)
Fruit juices	260.1 (255.9–264.5)	282.5^*^ (275.1–289.9)	247.5 (241.6–253.5)	265.0^*^ (258.2–271.7)
Milk and milk substitutes	234.2 (227.6–240.8)	250.7^*^ (243.3–258.0)	226.2 (219.5–232.9)	237.8^*^ (230.8–244.8)
Ultra-processed milk-based beverages	205.3 (192.0–218.7)	210.7 (197.1–224.3)	183.2 (159.3–207.1)	206.7 (188.2–225.1)

Household Budget Survey 2008–2009 and 2017–2018.

^*^p-value < 0.0001 for linear regression models, comparing differences between the surveys.

Regarding energy density, we saw an increase in high-calorie soft drinks, fruit refreshments, and milk and milk substitutes between the surveys, while coffee/tea and fruit juices decreased energy density intake (Table 4). Similar results were seen among men and women and all age groups, except for high-calorie soft drinks and fruit refreshments among individuals 60 years old or more and milk substitutes among men and individuals between 10 and 19 years old and between 40 and 59 years old. For fruit juices, the energy density decreased only among men and individuals between 20 and 39 years old (data not shown).

**Table 4:** Energy density (kcal/ml) and 95% confident interval (95% CI) of beverages groups, in 2008–2009 and 2017–2018.

**Beverage group**	**Brazil**	
	**2008–2009**	**2017–2018**
High-calorie soft drinks	36.4 (36.3–36.5)	37.4^*^ (37.1–37.6)
Fruit refreshments	28.8 (27.3–30.3)	32.5^*^ (31.1–33.8)
Alcoholic beverages	63.4 (59.8–67.0)	64.9 (61.4–68.4)
Coffees/teas	54.0 (53.4–54.7)	50.9^*^ (50.3–51.4)
Fruit juices	64.6 (64.0–65.2)	63.1^*^ (62.4–63.8)
Milk and milk substitutes	67.5 (66.7–68.3)	69.5^*^ (68.6–70.4)
Ultra-processed milk-based beverages	83.6 (82.1–85.0)	82.2 (81.4–83.1)

Household Budget Survey 2008–2009 and 2017–2018.

^*^p-value < 0.0001 for linear regression models, comparing differences between the survey.

## 4. Discussion

The assessment of changes in the portion sizes of beverages consumed in Brazil between 2008 and 2018 showed a change in the consumption of the population throughout the analyzed period. The individuals started to consume drinks less frequently, according to the occasion of consumption, but in larger quantities.

This decrease in the frequency of consumers and increase in the size of portions may be due to the offer of larger portions in commercial establishments, favoring the ingestion of excess beverages. This fact can be better understood by exposure to foods or beverages that are double or triple in size and, as it is a single package/portion, the individual believes that it is possible to consume it all at once (2).

The portion size effect (PSE) is the phenomenon, that, over time, is responsible for favoring the consumption of increasingly high-calorie portions. Some factors related to individual characteristics and hedonism and that intensify this process are of great importance to minimize the positive energy balance generated in the individual, especially in children. The high energy density, which tends to make the food/beverage more palatable, regardless of the caloric concentration, the consumer's BMI, directly proportional to how appetizing the food/beverage is, and the subjectivity in food choices are some of the considered points (15).

In the present study, it is possible to observe that the groups of alcoholic beverages, fruit refreshments, high-calorie soft drinks, milk and milk substitutes, and fruit juices were the groups that showed an increase in the portion sizes. The significant increase of 39% in the portion size of alcoholic beverages predominantly consumed by women aged between 20 and 40 years is worth mentioning, as it raises an alert for future excessive alcohol consumption, which may represent a public health problem. A study carried out by Bezerra and Alencar (13) documented that alcoholic beverages and soft drinks were responsible for the highest consumption of portions, and the intake of these groups was associated with excess weight in the Brazilian population.

According to the American Dietary Guidelines 2020–2025, the standard dose of alcohol is 14 g, which is equivalent to 355 ml of beer, 148 ml of wine and 45 ml of spirits. The limit dose per day, according to sex, is limited to one for women and two for men (16). In our study, the average intake of beer increased from 954.5 ml (95% CI: 879.6–1,029.4) in 2008–2009 to 1,190.4 ml (95% CI: 1085.7–1,295.2) in 2017–2018, among men. Among women, the average intake was 668.4 ml (95% CI: 580.7–756.1) and 921.9 (95% CI: 815.0–1,028.9), respectively. In both surveys, the average portion sizes were inappropriate amounts according to the recommendation. This is an important finding of our results, once the increase shows the worsening of this behavior.

For spirits and wines, there was no difference between the surveys among men (spirits: 201.4; 95% CI: 166.2–236.6 in 2008–2009 and 254.4; 95% CI: 214.0–294.7 in 2017–2018; wine: 272.0; 95% CI: 233.0–311.0 in 2008–2009 and 357.3; 95% CI: 246.0–468.6 in 2017–2018) and women (spirits: 257.5; 95% CI: 216.7–298.3 in 2008–2099 and 247.7; 95% CI: 202.0–293,3 in 2017–2018; wine: 249.3; 95% CI: 207.1–191.4 in 2008–2009 and 293.8; 95% CI: 234.0–353.5 in 2017–2018).

In addition to weight gain, high alcohol intake can stimulate risky behaviors, and cause stress intolerance and reproductive, thyroid and immunity dysfunctions. There are reports that excessive alcohol consumption together with a diet rich in ultraprocessed foods also increases the number of non-communicable chronic diseases, such as systemic arterial hypertension and overweight and obesity (17, 18).

The groups of fruit refreshments, high-calorie soft drinks and fruit juices were the beverages that showed the highest increase in portion size both in Brazil and among men and women. These drinks are often rich in free sugars, which in addition to providing low satiety to the body, stimulate excessive energy intake, contributing to the emergence of overweight, obesity, diabetes and cardiovascular disorders (2). North-American preschool children who were given 50% larger portions of food and milk than the usual portions for 5 days did not demonstrate self-regulation in response to excessive caloric intake from these large portions (19).

The conditioned food stimulus of larger portion sizes, generated by the available amount, especially since childhood, can cause the early consumption of these increased hypercaloric portions, which can lead to overweight and obesity. One way to minimize this exposure to high-calorie foods, given the relationship between providing larger portions, excessive consumption and increased body weight, is to reduce the portion size of high-calorie foods and develop products with lower energy density (15). In addition, the regularization of nutritional labeling is another factor that can minimize energy intake, since the information provided in a reliable way can influence the individual's choice (20).

Although a strong positive association between energy density and energy intake is observed, indicating that lower-energy dense diets may be efficacious for weight management, it is important to consider the level of beverage processing. One strategy of the food industry to decrease the energy content of beverages is substituting sugar for artificial sweeteners, which decreases daily energy intake and likely promotes weight loss (21). However, they undergo industrial handling with the inclusion of highly processed ingredients, such as artificial sweeteners, with harmful health outcomes. Some long-term prospective studies have raised the concern that the consumption of artificial sweeteners might cause alterations in the gut microbiome toward a more inflammatory pattern of gut microbiota and in metabolic pathways linked to glucose tolerance and dysbiosis in humans (22). A limitation of our study is that, in both surveys, we have limited information about the consumption of diet/light soft drinks.

Coffee/tea, fruit juices, and alcoholic beverages showed the highest energy density, after milk and milk substitutes and ultra-processed milk-based beverages. This finding must be related to the amount of sugar added to these beverages. In our survey, we asked which is the most frequent sweetener used to sweeten their beverages (sugar, artificial sweetener, both, or nothing). We estimated the amount of sugar in beverages based on their answers, considering 10% of sugar if the individuals used mostly sugar, 5% if the individuals used both sugar and artificial sweetener, and no addition with the person used only artificial sweetener or none. This approach can inflate energy content of beverages. However, it is important to consider that independent of the method applied to estimate sugar use, calories from sugar-containing and alcoholic beverages contain energy seem to be inadequately sensed by the satiety mechanisms in the gastrointestinal track which control appetite and body fat. These mechanisms are different for milk calories (23).

Studies about food energy density and its relationship with energy intake and body weight status have discussed different approaches to calculating dietary energy density, including or not beverages in ED estimation (24). Our study is focusing only on beverage intake, not being necessary to consider beverage inclusion criteria.

In terms of food portion size, both the seller and the consumer can share the responsibility for the commercialized/consumed portion, in an attempt to reduce the damage to individual and environmental health, since the excessive production of food and beverages causes unnecessary depletion and pollution to the planet (25).

High-calorie soft drinks and fruit refreshments were some of the most frequently consumed beverages in both surveys. Their consumption may be related to eating outside the home. A Brazilian study identified the foods most often purchased in establishments outside the home and found that soft drinks are the most frequently purchased beverages in snack bars and cafeterias (26). The sweetness, characteristic of these beverages, is a factor that influences the body's metabolic response since it interferes with the perception of sweet taste and the reward system (27). The level of processing, the way of preparation, the energy density, and nutritional content of beverages impact the homeostatic functioning of the metabolism and lead to excessive beverage intake (28). This deregulation tends to worsen with the increase in portion sizes (29).

One limitation of the present study is related to the difference in the tool for obtaining food consumption data, which in the 2008–2009 edition of HBS was the food record and in the 2017–2018 HBS was the 24-h recall. However, in both surveys the interviewers were trained to ask investigative questions and review all records and recalls together with the individual. The photographs with examples of food portions were the same in both surveys, minimizing errors in the estimates of the consumed amount.

We can highlight that this study is the first to assess differences in the portion sizes of beverages consumed in Brazil at a national level, contributing to the development of strategies that consider portion sizes in the prevention of the excessive consumption of calories and, consequently, weight gain and the development of chronic non-communicable diseases associated with a poor diet.

We observed a decrease in the frequency of beverage consumers between 2008 and 2018 in Brazil. The group of alcoholic beverages showed a substantial increase in the average of consumed portion size, followed by fruit refreshments, high-calorie soft drinks, milk and milk substitutes, and fruit juices. The coffee/tea group was the only one that showed a reduction in the size of the consumed portions. Our study presents an initial assessment of the evolution of the portion size of beverages consumed in a representative sample of the Brazilian population.

## Author's note

The present article originates from the dissertation by Jessica Brito Cavalcante, presented on December 9, 2021. The authors have a curriculum registered on the CNPq Lattes platform.

## Data availability statement

The datasets presented in this study can be found in online repositories. The names of the repository/repositories and accession number(s) can be found at: https://www.ibge.gov.br/.

## Ethics statement

The studies involving human participants were reviewed and approved by Comitê de Ética em Pesquisa da Universidade do Estado do Rio de Janeiro. Written informed consent from the participants' legal guardian/next of kin was not required to participate in this study in accordance with the national legislation and the institutional requirements.

## Author contributions

JC and TV participated in the analysis and interpretation of data and writing of the manuscript. RS coordinated the research, participated in the interpretation of data, and writing and final review of the manuscript. IB idealized the manuscript, participated in the analysis and interpretation of data, and writing and final review of the manuscript. All authors contributed to the article and approved the submitted version.
